# Pretreatment Neutrophil-to-Lymphocyte Ratio and Lactate Dehydrogenase Predict the Prognosis of Metastatic Cervical Cancer Treated with Combination Immunotherapy

**DOI:** 10.1155/2022/1828473

**Published:** 2022-10-18

**Authors:** Mingxia Cheng, Guiling Li, Zhongan Liu, Qin Yang, Yao Jiang

**Affiliations:** Cancer Center, Union Hospital, Tongji Medical College, Huazhong University of Science and Technology, Wuhan, China

## Abstract

**Background:**

Immune checkpoint inhibitors have considerably changed the treatment paradigm for metastatic cervical cancer; nonetheless, only a proportion of patients achieve a durable response. Therefore, exploring the predictive biomarkers of immunotherapy response is of crucial importance. This study aimed to evaluate the predictive and prognostic value of hematological parameters in patients with metastatic cervical cancer treated with combination immunotherapy.

**Methods:**

Clinical data of patients with metastatic cervical cancer treated with combination immunotherapy between June 2019 and April 2021 were retrospectively analyzed. Receiver operating characteristic curve analysis was performed to determine the cut-off values of continuous variables, and binary logistic analysis was conducted to compare the treatment response between groups. The Kaplan–Meier method was applied for survival analysis. A Cox proportional hazards regression model was used to identify factors associated with progression-free survival (PFS).

**Results:**

Seventy patients were included in this study. The cut-off values for the neutrophil-to-lymphocyte ratio (NLR) and lactate dehydrogenase (LDH) were 5.33 and 195.00 U/L, respectively. High pretreatment NLR (≥5.33) was correlated with decreased objective response rate (53.19% vs. 78.26%, *p* = 0.048). The survival analysis revealed that high pretreatment NLR (hazard ratio [HR] = 2.401, 95% confidence interval [CI]: 1.151–5.009, *p* = 0.020) and LDH level (HR = 1.987, 95% CI: 1.029–3.835, *p* = 0.041) were independent prognostic factors associated with short PFS.

**Conclusions:**

Our study suggested that high pretreatment NLR and LDH values were independently correlated with poor survival in patients with metastatic cervical cancer treated with combination immunotherapy. Pretreatment NLR and LDH values could serve as potential biomarkers that may aid in the selection of patients who would benefit from combination immunotherapy. Further prospective studies investigating the prognostic value of NLR and LDH are warranted. Trial registration number: UHCT22008.

## 1. Introduction

Cervical cancer ranks as the fourth leading cause of morbidity and mortality in women globally, with a 5-year survival rate of 18% in patients with metastatic disease [1, 2]. Recently, immune checkpoint inhibitors (ICIs) have considerably improved the treatment landscape for metastatic cervical cancer. PD-1 inhibitors, pembrolizumab and nivolumab, have been approved for second-line or subsequent therapy in metastatic cervical cancer with a modest response rate of approximately 15% [3, 4]. More recently, the combination of pembrolizumab and chemotherapy, plus or minus bevacizumab, has been approved for patients with metastatic cervical cancer [5]. However, only a proportion of patients achieve a durable response. Therefore, there is an urgent need to explore predictive biomarkers for immunotherapy efficacy.

Several predictive biomarkers for immunotherapy have been investigated in cervical cancer. Among them, PD-L1 expression, tumor mutational burden (TMB), and microsatellite instability (MSI) have been approved for selecting patients who would benefit from single-agent pembrolizumab [4, 6, 7]. However, the predictive role of these biomarkers in the context of combination immunotherapy is far from being clarified [8]. In the CHECKMATE-358 and CLAP studies, clinical efficacy was observed in patients treated with combination immunotherapy, irrespective of PD-L1 status [9, 10]. The predictive value of TMB and MSI for immunotherapy has also been reported [11, 12]. However, only 25% and 1.9% of patients with cervical cancer exhibit high TMB (≥10 mutations/Mb) or MSI, respectively [13]. Additionally, tumor tissue specimens are sometimes difficult to obtain, thus limiting the use of PD-L1, TMB, and MSI tests [14].

Recently, hematological parameters have been studied and exhibited potential for predicting immunotherapy efficacy. Routine blood tests have the advantage of being easily available and cost-effective. Increased neutrophil-to-lymphocyte ratio (NLR) or lactate dehydrogenase (LDH) predicted poor prognosis in patients treated with immunotherapy in various cancers [15–17]. In cervical cancer, elevated NLR and LDH levels have been reported to be correlated with adverse clinical outcomes [18–21]. However, previous studies mainly focused on the correlation between hematological parameters and clinical outcomes in patients with cervical cancer treated with conventional therapy, including surgery, chemotherapy, and radiotherapy [22–26]. The predictive value of hematological parameters in patients with cervical cancer treated with combination immunotherapy remains unclarified. Thus, this study was conducted to investigate the predictive and prognostic value of hematological parameters in patients with metastatic cervical cancer who underwent combination immunotherapy. The findings of this study may aid in identifying patients who would benefit most from combination immunotherapy.

## 2. Materials and Methods

### 2.1. Patients

Clinical data of patients with metastatic cervical cancer who underwent combination immunotherapy at the Cancer Center in the Union Hospital, Tongji Medical College, Huazhong University of Science and Technology (Wuhan, China) between June 2019 and April 2021 were reviewed. The inclusion criteria were as follows: (1) recurrent or metastatic cervical cancer, (2) treatment involving at least two cycles of combination immunotherapy, (3) at least one measurable lesion at baseline according to Response Evaluation Criteria in Solid Tumors (RECIST) version 1.1. Patients who underwent single-agent immunotherapy, withdrew immunotherapy because of intolerable toxicities, lacked follow-up data, or received drugs that improved blood cell function within 2 weeks from the first dose of immunotherapy were excluded from the analysis.

Baseline clinicopathological data, including age, histology, metastatic sites, lines of prior systemic treatment, and hematological parameters, were retrieved from medical records. The NLR refers to the absolute neutrophil count divided by the lymphocyte count measured in peripheral blood, whereas the platelet-to-lymphocyte ratio (PLR) refers to the platelet count divided by the lymphocyte count. The monocyte-to-lymphocyte ratio (MLR) refers to the monocyte count divided by the lymphocyte count.

This retrospective study was conducted in accordance with the principles embodied in the 1964 Declaration of Helsinki and was performed with the patients' understanding and consent. Furthermore, this study was approved by the Ethics Committee of Union Hospital, Tongji Medical College, Huazhong University of Science and Technology (approval no.: 20220023).

### 2.2. Follow-Up and Evaluation

All patients were regularly followed up until their death or the cut-off date for this study (October 20, 2021). Baseline tumor assessment was performed before immunotherapy initiation. Subsequently, the patients underwent imaging examination every 8–12 weeks. Clinical responses (complete response, partial response, stable disease, and progressive disease) were evaluated using the RECIST criteria version 1.1. The objective response rate (ORR) refers to the percentage of patients who achieved complete and partial responses. The disease control rate refers to the percentage of patients who achieved complete response, partial response, and stable disease. Progression-free survival (PFS) refers to the duration from the date of immunotherapy initiation to disease progression or until the last follow-up visit.

### 2.3. Statistical Analyses

Statistical analyses were performed using SPSS version 26.0 (IBM Corp., Armonk, NY, USA). For descriptive analysis, continuous and categorical variables were expressed as medians (range) and percentages, respectively. Receiver operating characteristic (ROC) curve analysis was performed to identify the cut-off values of continuous variables. The median value was regarded as the cut-off value if the area under the ROC curve was less than 0.50. Binary logistic analysis was conducted to analyze the correlation between clinical response and baseline characteristics. The Kaplan–Meier method was applied for survival analysis. A Cox proportional hazards regression model was used for univariate and multivariate analyses. A two-sided *p* value of < 0.05 was considered statistically significant.

## 3. Results

### 3.1. Clinical Characteristics and Treatment

Seventy patients with metastatic cervical cancer treated with combination immunotherapy between June 2019 and April 2021 were included in this study. The baseline demographic characteristics and hematological parameters of patients are presented in Table 1. The median patient age was 51 (29–77) years, and 54 (77.14%) patients had squamous cell carcinoma. The majority of patients (80.00%) previously received radiotherapy. Of these patients, 27 (38.57%) had only local recurrence, and 24 (34.29%) received two or more lines of systemic treatment before immunotherapy initiation. Of all patients, 21 (30.00%) were treated with a combination of PD-1 inhibitor plus chemotherapy, whereas 49 (70.00%) were treated with a combination of PD-1 inhibitor plus chemotherapy and antiangiogenic agents.

### 3.2. Relationship between Hematological Parameters and Clinical Response

Complete response, partial response, and stable disease were observed in 8 (11.43%), 35 (50.00%), and 13 (18.57%) patients, respectively. The ORR was 61.43%. The ROC curve analysis indicated that the cut-off values for NLR and LDH were 5.33 and 195.00 U/L, respectively. Patients with high NLR (≥5.33) showed lower ORR (53.19% vs. 78.26%, *p* = 0.048). The treatment responses based on baseline clinical characteristics are shown in Table 2. The PLR, MLR, and albumin-to-globulin ratio, as well as the levels of squamous cell carcinoma-associated antigen, LDH, and alkaline phosphatase, were not significantly associated with the clinical response.

### 3.3. Prognostic Factors for PFS

**Figure 1:**
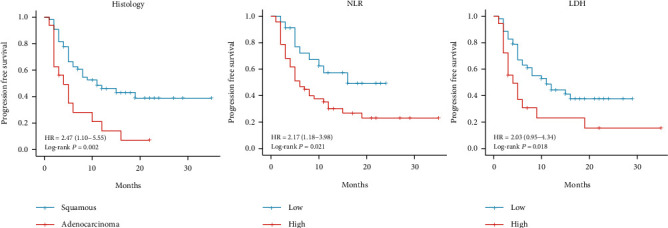
Kaplan–Meier curve for the progression-free survival of patients with cervical cancer who were grouped according to histology as well as pretreatment NLR and LDH values. HR: hazard ratio, NLR: neutrophil-to-lymphocyte ratio, and LDH: lactate dehydrogenase.

The median PFS of all patients was 8.0 months (95% confidence interval [CI]: 3.274–12.726). The univariate analysis showed that histology, NLR, and LDH level were significant prognostic factors for PFS. Patients with adenocarcinoma had shorter PFS than those with squamous carcinoma (hazard ratio [HR] = 2.448, *p* = 0.006). Furthermore, patients with high NLR (≥5.33) had shorter PFS (HR = 2.140, *p* = 0.035). Similarly, a high LDH (≥195.00 U/L) level was correlated with shorter PFS (HR = 2.073, *p* = 0.026). The multivariate analysis revealed that histology of adenocarcinoma (HR = 3.258, 95% CI: 1.652–6.422, *p* = 0.001), high NLR (HR = 2.401, 95% CI: 1.151–5.009, *p* = 0.020), and high LDH level (HR = 1.987, 95% CI: 1.029–3.835, *p* = 0.041) remained as independent predictive factors for inferior PFS (Table 3). The Kaplan–Meier curve for the PFS of patients grouped according to histology as well as pretreatment NLR and LDH values are presented in Figure 1.

## 4. Discussion

An increasing number of patients with metastatic cervical cancer are being treated with immunotherapy in clinical practice; however, only a proportion of patients achieve a durable response. Therefore, an investigation of the predictive biomarkers of immunotherapy response is important. Recently, several studies have shown that hematological parameters can predict immunotherapy response in various cancers [27–29]. Nevertheless, information on the prognostic value of routine blood parameters in cervical cancer after immunotherapy is scarce. In this study, we assessed the baseline hematological parameters in patients with metastatic cervical cancer who underwent combination immunotherapy and confirmed that high pretreatment NLR and LDH values were associated with inferior PFS.

Neutrophils, which play essential roles in tumor development and progression, can be mechanistically recruited to the tumor microenvironment, secrete proliferative factors, and suppress T-lymphocyte activity, thereby promoting tumor angiogenesis, invasion, and metastasis [30–33]. The NLR is a systemic inflammation indicator of the balance between antitumor immune response and protumor inflammation and is an independent prognostic biomarker in various malignancies [16, 19, 34, 35]. Lima et al. reported an adverse association between NLR and prognosis in cervical cancer [36], whereas Zhang et al. showed the correlation of preoperative NLR with unfavorable histopathological features and prognosis in patients with cervical cancer who underwent surgery [24]. Additionally, pretreatment NLR could be a prognostic factor after chemotherapy and radiotherapy for cervical cancer [19, 22, 37]. More recently, the adverse association between NLR and prognosis in patients treated with immunotherapy has been reported. Dharmapuri et al. reported that both pretreatment and posttreatment NLR ≥ 5 was correlated with inferior overall survival (OS) in patients with hepatocellular carcinoma after immunotherapy [15]. In a cohort comprising 175 patients with non-small-cell lung cancer (NSCLC) treated with nivolumab, pretreatment NLR ≥ 5 was predictive of poor PFS and OS [38]. Nevertheless, the predictive role of NLR in cervical cancer treated with combination immunotherapy has not been investigated. The results of our study indicated that pretreatment NLR ≥ 5.33 was significantly associated with worse ORR and was independently predictive of inferior PFS in patients with metastatic cervical cancer after combination immunotherapy. The cut-off value for NLR was similar to the value reported in the aforementioned studies.

LDH is a key metabolic enzyme involved in the glycolytic pathway, which converts pyruvate to lactate, thereby causing a hypoxia-inducible factor 1a cascade, establishing a hypoxic tumor microenvironment and promoting tumor angiogenesis and aggressiveness. Elevated LDH level correlates with high tumor burden and poor survival in various malignancies [39–41]. Li et al. observed that the serum LDH level was elevated in most patients with cervical cancer and was strongly correlated with adverse outcomes [23]. Wang et al. revealed that high preradiotherapy LDH level was associated with poor OS in patients with cervical cancer who underwent chemoradiotherapy [18]. An inverse correlation between baseline LDH level and survival outcomes has recently been reported in patients treated with immunotherapy [42, 43]. A single-center retrospective study that included 366 patients with NSCLC who received ICI monotherapy had confirmed that a low LDH level indicated superior OS [44]. Another cohort study involving 153 patients with solid tumors treated with ICI monotherapy or combination therapy showed that elevated LDH level was associated with poor prognosis, irrespective of tumor types [17]. However, the predictive role of LDH after combination immunotherapy in cervical cancer remains unclear. In this study, we showed that baseline LDH level ≥ 195.00 U/L was correlated with inferior PFS in patients with cervical cancer treated with combination immunotherapy. Notably, no consensus regarding the cut-off value for the LDH level has been reached in previous studies. A meta-analysis showed the correlation between high LDH level and inferior survival in several malignancies, and there was no correlation between the LDH cut-off value and the reported hazard risk for OS [45].

To our knowledge, this study is the first to explore the correlation between hematological parameters and combination immunotherapy efficacy in patients with cervical cancer. Our analysis demonstrated that high pretreatment NLR and LDH values predicted poor prognosis in patients with metastatic cervical cancer who underwent combination immunotherapy. The findings of our study may aid in the early identification of patients with metastatic cervical cancer who would benefit from combination immunotherapy.

This study had some limitations. First, it is a retrospective study with a relatively small number of patients, which might have led to selection bias. Second, other inflammation-related peripheral blood indicators, such as C-reactive protein, were not analyzed in this study because only a proportion of patients were tested for it. Therefore, further investigations involving other inflammation markers derived from peripheral blood are required to determine their prognostic significance. Finally, the dynamic change in hematological parameters, which may reflect the dynamic change in the balance between cancer-associated inflammation and host immune response, should be investigated. Considering these limitations, well-designed, multicenter, prospective studies are warranted.

## 5. Conclusions

Our study indicated that high baseline NLR and LDH values were correlated with poor survival in patients with metastatic cervical cancer who underwent combination immunotherapy. Pretreatment NLR and LDH values could serve as potential biomarkers that may aid in the early identification of patients who would benefit from combination immunotherapy. Further prospective studies are necessary to validate the clinical application of our findings.

## Figures and Tables

**Table 1:** General characteristics of patients with metastatic cervical cancer treated with combination immunotherapy.

Variables	*N* = 70 (%)
Median age, years (range)	51 (29–77)
Histology	
Squamous cell carcinoma	54 (77.14)
Adenocarcinoma or others	16 (22.86)
Disease status	
Local recurrence	27 (38.57)
Distant metastasis	43 (61.43)
Prior radiotherapy	
Yes	56 (80.00)
No	14 (20.00)
Line of immunotherapy	
First-line or second-line	46 (65.71)
Third-line or more	24 (34.29)
NLR	5.17 (3.19–9.16)
PLR	270.50 (174.19–363.49)
MLR	0.51 (0.31–0.78)
SCC-Ag (ng/mL)	2.85 (0.90–14.10)
AGR	1.50 (1.30–1.63)
LDH (U/L)	166.50 (146.75–195.75)
ALP (U/L)	87.50 (70.00–106.00)

Abbreviations: AGR, albumin-to-globulin ratio; ALP, alkaline phosphatase; LDH, lactate dehydrogenase; MLR, monocyte-to-lymphocyte ratio; NLR, neutrophil-to-lymphocyte ratio; PLR, platelet-to-lymphocyte ratio; SCC-Ag, squamous cell carcinoma-associated antigen.

**Table 2:** Correlation between the objective response rate and clinicopathological factors in patients with cervical cancer treated with combination immunotherapy.

Variables	ORR (%)	OR (95% CI)	*p*
Age (<50 vs. ≥50 years)	68.75 vs. 55.26	0.561 (0.210–1.502)	0.250
Histology (squamous cell carcinoma vs. adenocarcinoma)	68.52 vs. 43.75	0.389 (0.125–1.214)	0.104
Disease status (local recurrence vs. distant metastasis)	59.26 vs. 62.79	1.160 (0.433–3.109)	0.768
Prior radiotherapy (yes vs. no)	58.93 vs. 71.43	1.742 (0.486–6.241)	0.394
Line of immunotherapy (<3 vs. ≥3)	69.57 vs. 45.83	0.370 (0.134–1.026)	0.056
NLR (<5.33 vs. ≥5.33)	78.26 vs. 53.19	0.316 (0.100–0.991)	0.048
PLR (<297.92 vs. ≥297.92)	70.00 vs. 58.00	0.592 (0.195–1.794)	0.354
MLR (<0.29 vs. ≥0.29)	80.00 vs. 56.36	0.323 (0.082–1.274)	0.107
SCC-Ag (<2.85 vs. ≥2.85)	68.57 vs. 54.29	0.544 (0.205–1.444)	0.222
AGR (<1.80 vs. ≥1.80)	56.90 vs. 83.33	3.788 (0.761–18.849)	0.104
LDH (<195.00 vs. ≥195.00)	67.31 vs. 44.44	0.389 (0.130–1.162)	0.091
ALP (<87.50 vs. ≥87.50)	57.14 vs. 65.71	1.437 (0.547–3.781)	0.462

Abbreviations: AGR, albumin-to-globulin ratio; ALP, alkaline phosphatase; CI, confidence interval; LDH, lactate dehydrogenase; MLR, monocyte-to-lymphocyte ratio; NLR, neutrophil-to-lymphocyte ratio; OR, odds ratio; ORR, objective response rate; PLR, platelet-to-lymphocyte ratio; SCC-Ag, squamous cell carcinoma-associated antigen.

**Table 3:** Univariate and multivariate analyses of progression-free survival in patients with metastatic cervical cancer treated with combination immunotherapy.

Variables	Univariate		Multivariate	
	HR (95% CI)	*p*	HR (95% CI)	*p*
Age (<50 vs. ≥50 years)	1.198 (0.658–2.184)	0.554		
Histology (squamous cell carcinoma vs. adenocarcinoma)	2.448 (1.291–4.645)	0.006	3.258 (1.652–6.422)	0.001
Disease status (local recurrence vs. distant metastasis)	0.581 (0.320–1.053)	0.074		
Prior radiotherapy (yes vs. no)	0.611 (0.258–1.447)	0.263		
Line of immunotherapy (<3 vs. ≥3)	1.592 (0.865–2.930)	0.135		
NLR (<5.33 vs. ≥5.33)	2.140 (1.005–4.342)	0.035	2.401 (1.151–5.009)	0.020
PLR (<297.92 vs. ≥297.92)	1.614 (0.814–3.200)	0.171		
MLR (<0.29 vs. ≥0.29)	2.528 (0.993–6.431)	0.052		
SCC-Ag (<2.85 vs. ≥2.85)	1.218 (0.672–2.208)	0.516		
AGR (<1.80 vs. ≥1.80)	1.122 (0.537–2.345)	0.759		
LDH (<195.00 vs. ≥195.00)	2.073 (1.090–3.942)	0.026	1.987 (1.029–3.835)	0.041
ALP (<87.50 vs. ≥87.50)	0.973 (0.538–1.762)	0.929		

Abbreviations: AGR, albumin-to-globulin ratio; ALP, alkaline phosphatase; CI, confidence interval; HR, hazard ratio; LDH, lactate dehydrogenase; MLR, monocyte-to-lymphocyte ratio; NLR, neutrophil-to-lymphocyte ratio; PLR, platelet-to-lymphocyte ratio; SCC-Ag, squamous cell carcinoma-associated antigen.

## Data Availability

The data used to support the findings of this study are available from the corresponding author upon request.

## Abbreviations

CI: Confidence interval
HR: Hazard ratio
ICI: Immune checkpoint inhibitor
LDH: Lactate dehydrogenase
MLR: Monocyte-to-lymphocyte ratio
MSI: Microsatellite instability
NLR: Neutrophil-to-lymphocyte ratio
NSCLC: Non-small-cell lung cancer
ORR: Objective response rate
OS: Overall survival
PFS: Progression-free survival
PLR: Platelet-to-lymphocyte ratio
RECIST: Response evaluation criteria in solid tumors
ROC: Receiver operating characteristic
TMB: Tumor mutational burden.
